# Histone demethylase RBP2 induced by Helicobactor Pylori CagA participates in the malignant transformation of gastric epithelial cells

**DOI:** 10.18632/oncotarget.2185

**Published:** 2014-07-08

**Authors:** Xiuming Liang, Jiping Zeng, Lixiang Wang, Li Shen, Shuyan Li, Lin Ma, Xinyu Ci, Jingya Yu, Mutian Jia, Yundong Sun, Zhifang Liu, Shili Liu, Wenjuan Li, Han Yu, Chunyan Chen, Jihui Jia

**Affiliations:** ^1^ Department of Microbiology/Key Laboratory for Experimental Teratology of Chinese Ministry of Education, School of Medicine, Shandong University, Jinan, PR China; ^2^ Department of Biochemistry, School of Medicine, Shandong University, Jinan, PR China; ^3^ Department of Pharmacology, School of Medicine, Shandong University, Jinan, PR China; ^4^ Department of Hematology, Qilu Hospital, Shandong University, No.107,Wenhua Xi Road, Jinan 250012, Shandong, P. R. China

**Keywords:** RBP2, CagA, GC, Sp1, malignant transformation

## Abstract

Gastric epithelial cell malignant transformation induced by Helicobactor Pylori contributes to tumor development, but the underlying mechanisms for this remain unclear. Here we demonstrate that RBP2, a newly identified histone demethylase, can be induced by CagA via PI3K/AKT-Sp1 pathway depending on AKT phosphorylation. Sp1 directly binds to RBP2 promoter and enhances its expression then the upregulated RBP2 significantly increases Cyclin D1 transcription, which contributes to gastric epithelial cell malignant transformation. Further data indicate that knockdown of endogenous RBP2 dominantly inhibits gastric cancer (GC) development both in vitro and in vivo. In conclusion, this CagA- PI3K/AKT-Sp1-RBP2-Cyclin D1 pathway may serve as a novel mechanism for gastric epithelial cell malignant transformation and then gastric cancer (GC). Therefore, RBP2 may link chronic inflammation to tumor development and its inhibition may have potential therapeutic advantages.

## INTRODUCTION

There are 1,000,000 new gastric cancer (GC) patients per year in the world and GC ranks third among different kinds of tumors in China. Thus, it poses a great thread to the health of people worldwide and it is urgent to decrease its morbidity. Many factors, either chemical or infectious ones, contribute to the development of GC [1, 2]. It is well recognized that Helicobactor Pylori is the Class A oncogenic factor of GC [3]. Helicobactor Pylori infection causes chronic inflammation of gastric mucosa and if left uncontrolled, it will progress to dysplasia, which is a kind of stage of precancerous lesion [4]. And then the gastric epithelial cells in this stage will subject to malignant transformation, which results in the development of gastric cancer (GC). This kind of malignant transformation is characterized by uncontrolled epithelial cell proliferation, which is one of the major hallmarks of cancer [5]. Some canonical signaling pathways are involved in this process, including NF-κB and AKT/PI3K pathways. For instance, Helicobacter pylori CagA activates NF-κB by targeting TAK1 for TRAF6-mediated Lys 63 ubiquitination [6]; CagA(+) H. pylori induces Akt1 phosphorylation and inhibits transcription of p21(WAF1/CIP1) and p27(KIP1) via PI3K/Akt1 pathway [7]. However, the specific mechanism through which Helicobactor Pylori infection leads to tumor has not been fully elucidated.

Mounting data indicate that epigenetic molecules participate in the development of cancer, acting as oncogenes or tumor suppressors [8-10]. It was reported that epigenetic silencing through DNA and histone methylation of fibroblast growth factor receptor 2 exists in neoplastic pituitary cells [11]; MicroRNA-antagonism regulates breast cancer stemness and metastasis via TET-family-dependent chromatin remodeling [12]. In our previous work, we found the high expression of RBP2 in gastric cancer (GC) as well as in hepatocellular carcinoma (HCC) [13-15], and its inhibition can trigger cell senescence. Others found that loss of the retinoblastoma binding protein 2 (RBP2) histone demethylase suppresses tumorigenesis in mice lacking Rb1 or Men1 and Sharma SV published data illustrating a chromatin-mediated reversible drug-tolerant state in cancer cell subpopulations [16, 17]. Thus, we aim to investigate whether there is any relation between Helicobactor Pylori infection and RBP2 expression during the process of gastric epithelial cell malignant transformation which is closely connected with GC development.

In the present work, we find that RBP2 can be induced by Helicobactor Pylori virulence factor CagA, which is PI3K/AKT-Sp1 pathway dependent. The activated AKT by CagA enhances the expression of Sp1 and the latter binds to the promoter of RBP2 to simulate its expression. In addition, the upregulated RBP2 significantly contributes to the transcription of Cyclin D1, which eventually promotes gastric epithelial cell malignant transformation. Knockdown of endogenous RBP2 with specific small interference RNA dominantly inhibits GC cell proliferation in vitro and tumor development in vivo. RBP2 serves as an important bridge that links infection (inflammation) to cancer development, which, at least in part, delineates the mechanism for GC development induced by Helicobactor Pylori. Therefore, RBP2 inhibition may be a new strategy for the prevention of GC development.

## RESULTS

### RBP2 is overexpressed in GC cell lines and its inhibition suppressed cell proliferation in vitro and tumor development in vivo

**Figure1:**
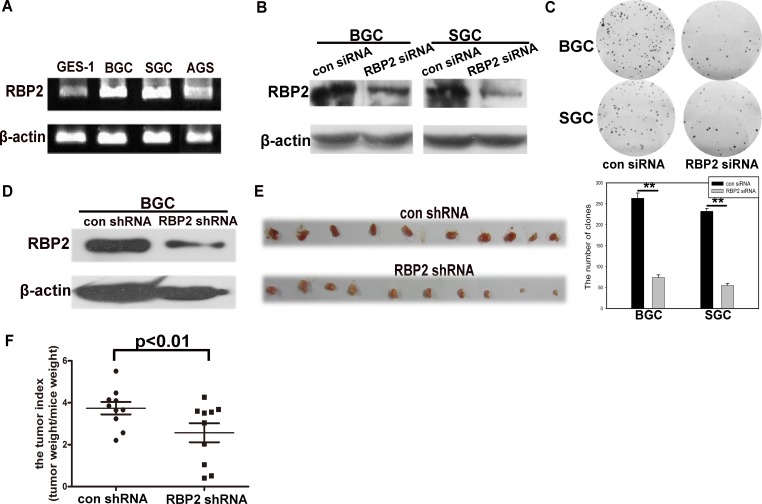
RBP2 is overexpressed in GC cell lines and its inhibition suppressed cell proliferation in vitro and tumor development in vivo (A) RBP2 mRNA expression in GC cell lines using Semi-quantitative PCR. (B) and (C) RBP2 protein expression and foci formation in RBP2 siRNA and the matched control siRNA treated GC cell lines respectively. Representative images are shown here from three independent biological replicates. (D) RBP2 protein expression in RBP2 shRNA and the matched control shRNA BGC cells. (E) Tumors formed in con shRNA and RBP2 shRNA group were harvested and photographed. (F) Tumor index in con shRNA and RBP2 shRNA group.

First, using GES-1 cell line, the immortal epithelial cells, as a control, we found the high expression of RBP2 in GC cell lines, especially in SGC-7901 and BGC-823 cells (Figure 1A, Figure S1A). Combing with the fact that RBP2 was overexpressed in GC tissues [13], we want to know whether it was involved in GC development. For this purpose, we decreased RBP2 expression by siRNA in SGC-7901 and BGC-823 cells and cell proliferation was significantly inhibited with RBP2 suppression (Figure 1B and 1C). Next, we constructed the BGC-823 cell line that can stably express RBP2 shRNA which inhibited RBP2 expression and the matched control cell line (Figure 1D), and then the above cells were injected subcutaneously into nude mice (2×10^5^/mouse). Half a month later, we harvested the formed tumors and photographed them. As can be seen in Figure 1E, tumors formed in RBP2 shRNA group were obviously smaller than that in control group. Furthermore, tumor index (tumor weight/mice weight) in RBP2 shRNA group was significantly lower than that in its control group (Figure 1F). These data strongly supported that RBP2 was critical for cell proliferation and tumor development in vitro and in vivo respectively, but the reason for the overexpressed RBP2 in GC tissues and GC cell lines was not understood.

### Helicobactor Pylori CagA can stimulate RBP2 expression and depletion of RBP2 attenuates cell proliferation induced by CagA

**Figure 2:**
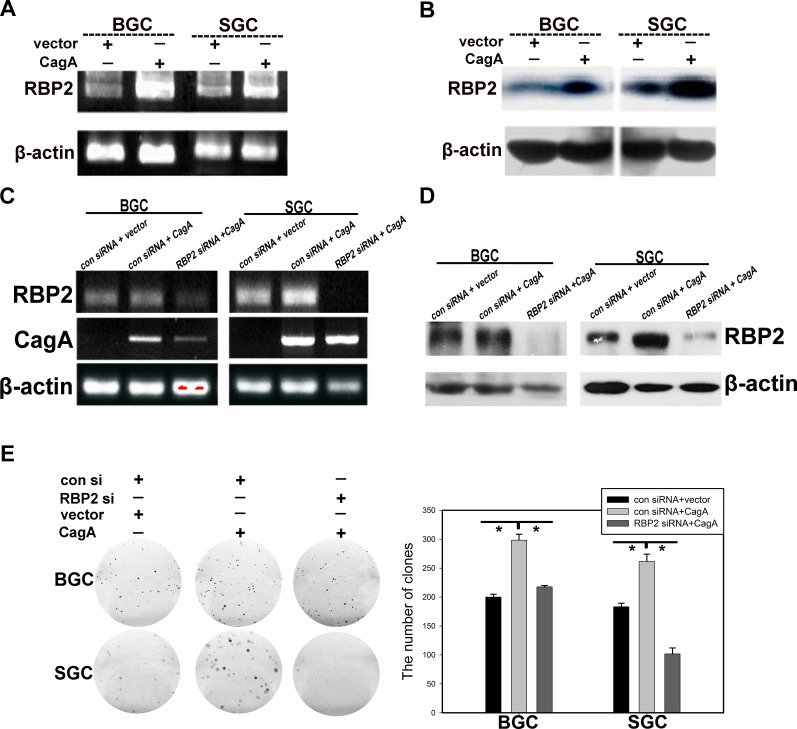
Helicobactor Pylori CagA can stimulate RBP2 expression and depletion of RBP2 abrogates cell proliferation induced by CagA (A) and (B) RBP2 expression at RNA and protein level with CagA transfection respectively. (C) and (D) Gene expression in RBP2 siRNA and CagA treatment solely or jointly GC cells at RNA level and protein level respectively. (E) Foci formation in RBP2 siRNA and CagA treatment solely or jointly GC cells. Representative images are shown here from three independent biological replicates.

Helicobactor Pylori infection can cause chronic inflammation of gastric mucosa and this can progress to dysplasia stage without Helicobactor Pylori eradication. Injected by type IV secretory system into host cells, CagA is the main virulence factor of Helicobactor Pylori [18]. Thus, we aim to identify whether there is any relationship between CagA injection and RBP2 expression during GC development. It can be seen from Figure 2A and 2B that CagA significantly enhanced RBP2 expression at both the mRNA and protein level. In addition, we inhibited RBP2 expression with RBP2 siRNA first and then transfected CagA into BGC-823 and SGC-7901 cells. Expectedly, RBP2 siRNA treatment relieved RBP2 upregulation induced by CagA at both mRNA and protein level(Figure 2C and 2D). Interestingly, depletion of RBP2 attenuated cell proliferation of GC cells induced by CagA (Figure 2E), which indicated that RBP2 was crucial, at least in part, for the pathogenicity conferred by Helicobactor Pylori.

### RBP2 expression induced by CagA is Sp1 dependent

**Figure 3:**
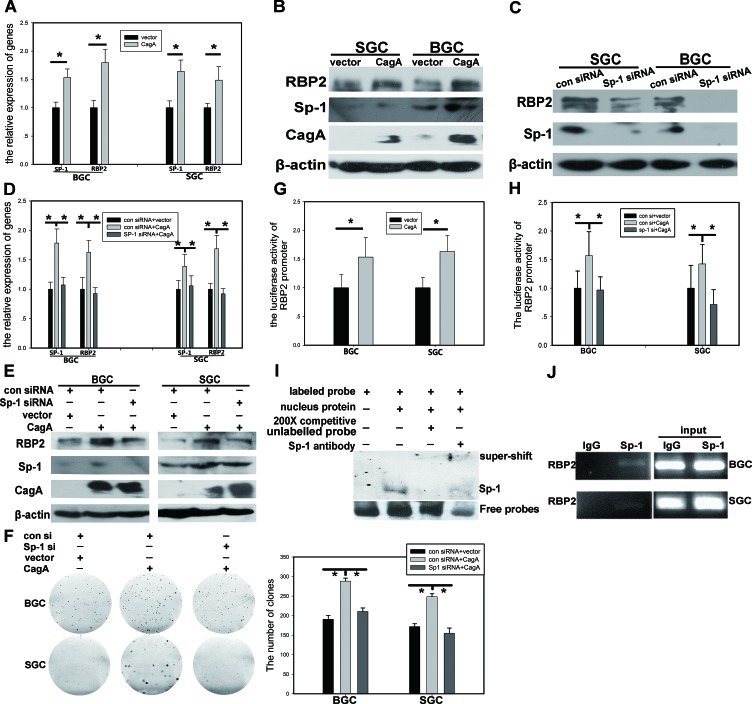
RBP2 expression induced by CagA is Sp1 dependent (A) and (B) RBP2 and Sp1 changes at RNA level and protein level with CagA transfection in GC cells was determined by QRT-PCR and western blot respectively. Data are mean±SD of 3 biological replicates, *: *P* <0.05 compared with negative control. (C) RBP2 and Sp1 protein changes with Sp1 siRNA treatment in GC cells. (D), (E) and (F) RBP2 and Sp1 changes at RNA level and protein level and foci formation with Sp1 siRNA and CagA treatment solely or jointly in GC cells. Data are mean±SD of 3 biological replicates, *: *P* <0.05 compared with negative control. (G) RBP2 promoter activity changes with CagA transfection in GC cells. Data are mean±SD of 3 biological replicates, *: *P* <0.05 compared with control. (H) RBP2 promoter activity changes with Sp1 siRNA and CagA treatment solely or jointly in GC cells. Data are mean±SD of 3 biological replicates, *: *P* <0.05 compared with control. (I) EMSA assay for the binding of Sp1 to RBP2 promoter. (J) ChIP assay for the binding of Sp1 to RBP2 promoter in GC cells.

RBP2 can be induced by CagA, but one problem existed. That is CagA is within the cytoplasm while RBP2 is mainly in the nucleus, so how can CagA has regulatory effect on RBP2? To tackle with this problem, we should find a molecule that can link CagA to RBP2. Hence, we analyzed RBP2 promoter to find some conservative elements that can be bound by common transcriptional factors. Fortunately, we found that the classical elements GGCGGG (Figure S1C) that can be recognized by Sp1 existed in RBP2 promoter [19]. Combining with the published data that Sp1 can be activated by Helicobactor Pylori infection [20], we next focused our investigation on regulation of RBP2 by Sp1. First, we transfected CagA into SGC-7901 and BGC-823 cells and RBP2, coupled with Sp1, was significantly induced at mRNA and protein level (Figure 3A and 3B). In addition, knockdown of Sp1 in GC cells dominantly reduced RBP2 expression (Figure 3C). Furthermore, depletion of Sp1 expression abrogated the induction of RBP2 by CagA in GC cells and the pro-proliferation effect of CagA was also relieved with Sp1 suppression (Figure 3D, 3E and 3F and Figure S1B). These data indicated that RBP2 induction by Helicobactor Pylori CagA was Sp1 dependent. To further testify if Sp1 directly acted on RBP2 promoter, we constructed RBP2 promoter plasmid (pRBP2-promoter) that contained GGCGGG element which can be bound by Sp1. As we can see from Figure 3G, co-transfection of CagA and pRBP2-promoter increased luciferase activity in GC cells and depletion of Sp1 expression relieved luciferase activity increase in these cells (Figure 3H). Furthermore, we performed EMSA experiment to confirm the binding of Sp1 protein to GGCGGG element within RBP2 promoter. The result indicated that Sp1 can specifically bind to GGCGGG element due to the fact that 200 times cold unlabeled probes competed with the labeled probes' binding (Figure 3I). In addition, super-shift band in the fourth lane verified the success of this experiment. At last, to make sure whether Sp1 directly binds to RBP2 promoter, ChIP was performed and we got the positive results (Figure 3J).

### PI3K/AKT participates in RBP2 induction by CagA through Sp1

**Figure 4:**
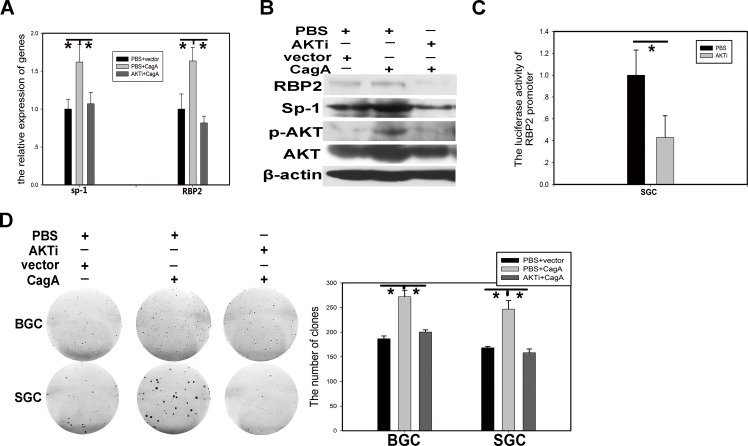
PI3K/AKT participates in RBP2 induction by CagA through Sp1 (A) and (B) RBP2 and Sp1 changes at RNA level and protein level with AKTi and CagA treatment solely or jointly in GC cells respectively. Data are mean±SD of 3 biological replicates, *: *P* <0.05 compared with control. (C) RBP2 promoter activity changes with AKTi treatment in SGC-7901 cells. Data are mean±SD of 3 biological replicates, *: *P* <0.05 compared with negative control. (D)Foci formation in GC cells with AKTi and CagA treatment solely or jointly. Representative images are shown here from three independent biological replicates.

We know that Helicobactor Pylori CagA can activate many canonical signaling pathways, but which one is involved in RBP2 induction via Sp1? Published data reported that Sp1 was closely associated with PI3K/AKT pathway and this pathway can also be activated by CagA [21, 22], so we wanted to link them together. In SGC-7901 cells, RBP2 and Sp1 induction at mRNA and protein level by CagA can be relieved when pretreating with AKT inhibitor (LY294002) (Figure 4A and 4B). In addition, RBP2 promoter luciferase activity was reduced by AKT inhibitor treatment in SGC-7901 cells (Figure 4C). GC cell proliferation stimulated by CagA was also relieved by AKT inhibitor (Figure 4D). These results demonstrated that PI3K/AKT pathway was critical for the link between CagA and Sp1 and then RBP2.

### Cyclin D1 is a downstream target of RBP2 and it confers the pro-proliferation effect on GC cells

**Figure 5:**
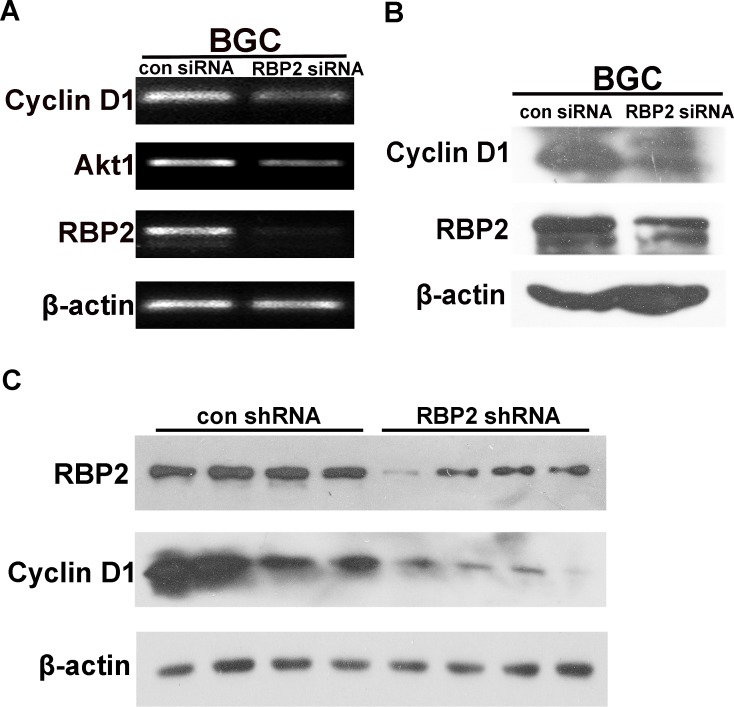
Cyclin D1 is a downstream target of RBP2 and it imposes the pro-proliferation effect (A) and (B) Cyclin D1 changes at RNA level and protein level with RBP2 siRNA treatment in BGC-823 cells respectively. (C) Western blot shows RBP2 and Cyclin D1 protein expression in tissues from nude mice in con shRNA and RBP2 shRNA group respectively.

RBP2 belongs to the regulatory molecules family which have downstream targets, so we need to find an effective molecule downstream RBP2. This molecule should be associated with cell cycle since tumorigenesis is closely related with uncontrolled cell proliferation. Published data showed that Cyclin D1 was a downstream target of RBP2 in lung cancer [23], so we tried to testify this in GC. As can be seen in Figure 5A and 5B, when RBP2 was inhibited in BGC-823 cells, Cyclin D1 expression decreased both at mRNA and protein level, which was in line with the finding in lung cancer. CagA stimulated RBP2 expression and the latter enhanced Cyclin D1 transcription, which contributed to the switch from inflammation to cancer development. To further confirm the regulation of Cyclin D1 by RBP2 in vivo, we performed western blot for them from tissues of tumors formed in RBP2 shRNA group and the matched control group as previously described. Notably, RBP2, as well as Cyclin D1 expression was decreased in RBP2 shRNA group compared with control group (Figure 5C), which implicated RBP2 may regulate Cyclin D1 expression in vivo.

### Co-expression of RBP2 and Sp1 in clinical samples from chronic inflammation to dysplasia

**Figure 6:**
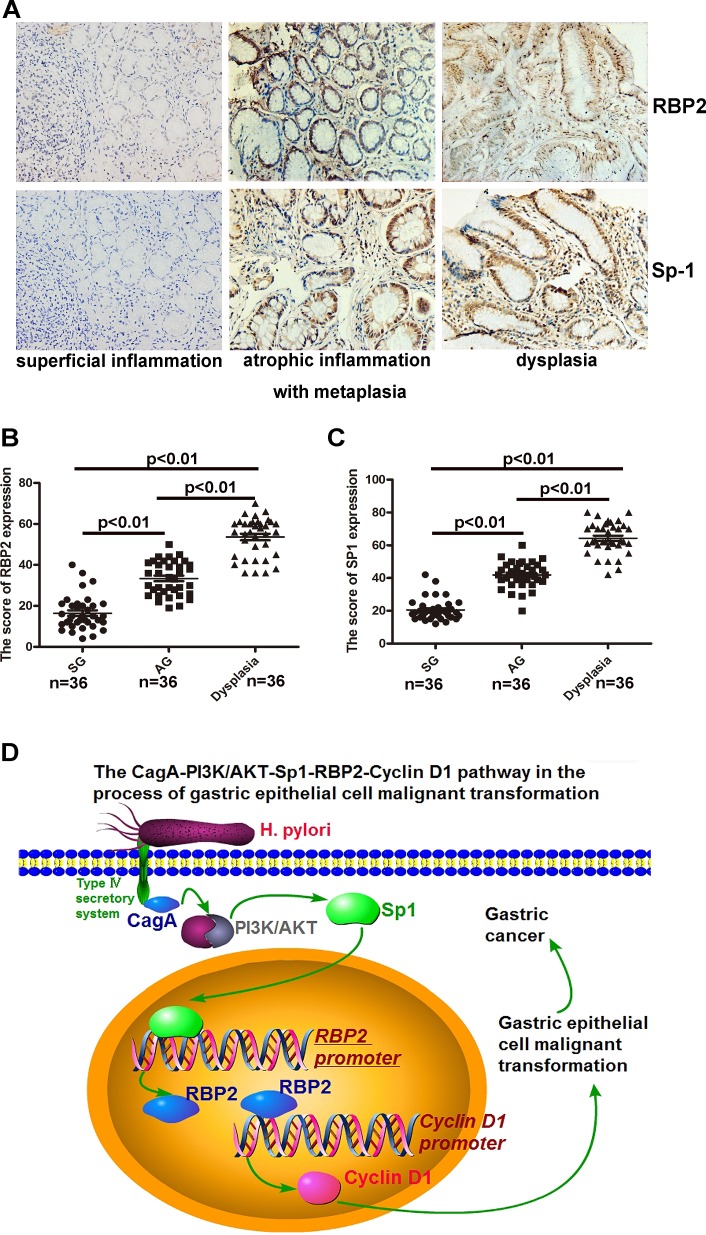
Co-expression of RBP2 and Sp1 expression in clinical samples from chronic inflammation to dysplasia (A) RBP2 and Sp1 expression in serial sections from tissues of clinical samples (chronic inflammation to dysplasia). Representative images are shown here. Original magnification, ×10. (B) and (C) A summary of RBP2 and Sp1 expression in the above tissues. (D) An outline of the main pathways illustrated in our work.

We collected 36 clinical samples of superficial inflammation, atrophic inflammation with metaplasia and dysplasia respectively. IHC staining for RBP2 and Sp1 in serial sections indicated their co-expression in clinical samples and their expression gradually increased with progression of disease (Figure 6A, 6B and 6C). Thereby RBP2 and Sp1 were positively related during the process of gastric epithelial cell malignant transformation, which confirmed our previous results in vitro.

## DISCUSSION

It is a complicated process from inflammation to tumor development, which involves many canonical signaling pathways [24-26], as well as epigenetic modification [27]. Here, we present data that RBP2 can be induced by Helicobactor Pylori CagA through AKT-Sp1 pathway and Sp1 directly binds to RBP2 promoter, contributing to its induction during infection. The upregulated RBP2 then stimulates Cyclin D1 transcription, which promotes malignant transformation of gastric epithelial cells. This CagA-PI3K/AKT-Sp1-RBP2-Cyclin D1 pathway may be a novel mechanism for the switch from inflammation to GC development and the key bridge molecule RBP2 may have potential therapeutic advantages.

It is well established that chronic inflammation of gastric epithelium contributes, in most cases, to tumor development, during which gastric epithelial cells undergo malignant transformation characterized by sustained cell proliferation. Therefore, Helicobactor Pylori is thought as a Class A oncogenic factor of GC for the reason that its infection gives rise to chronic inflammation of gastric epithelium if left uncontrolled. Increasing data are focusing on the switch from chronic inflammation to tumor and this kind of inflammation is defined as nonresolving inflammation which is universal in the precancerous lesion stage of many tumors, including GC, HCC, clonal carcinoma and so on [28, 29]. What we need to know are the factors that promote epithelial malignant transformation and much attention is paid to the investigation of traditional transcriptional factors and canonical signaling pathways. However, accumulating data indicated that epigenetic modification which imposed regulation effects on significant biological processes, either physiological or pathogenic ones, is of equal importance. More often than not, epigenetic modification includes DNA methylation, histone modification, chromatin remodeling and non-coding RNAs' regulation. In the present work, we are focusing on RBP2, a newly identified H3K4 demethylase [30-32], since it is closely associated with tumor development, but the role RBP2 plays in the the switch from chronic inflammation to tumor remains unclear. From the results we got, we find the novel CagA-PI3K/AKT-Sp1-RBP2-Cyclin D1 pathway that links chronic inflammation to tumor during GC development. As an epigenetic molecule, RBP2 can be induced by CagA and is involved in the canonical PI3K/AKT signaling pathway. In addition, we previously reported that some CDKIs (cyclin dependent kinase inhibitors, such as p16^ink4a^, p21^CIP2^ and p27^kip1^) were negatively regulated by RBP2 [13], thus, it was conceivable that the upregulated RBP2 by CagA suppressed CDKIs expression, facilitating bypass of cell senescence and contributing to uncontrolled cell proliferation. Interestingly, our data also indicated that RBP2 appeared to have feedback regulation on AKT, so we will focus on this investigation in the following projects. In addition, apart from RBP2, other key epigenetic molecules also appear to have an impact on the regulation of this kind of switch. Herein more work needs to be done to elucidate the underlying mechanisms to enrich the understanding of tumor development.

In summary, for the first time, we discovered the CagA-PI3K/AKT-Sp1-RBP2-Cyclin D1 pathway that contributed to the switch from chronic inflammation to tumor in GC development. RBP2 acted as a bridge that linked infection (nonresolving inflammation) of Helicobactor Pylori to epithelial cell malignant transformation, which finally led to tumor (uncontrolled cell proliferation). RBP2 may have potential therapeutic advantages for the prevention of tumor development.

## METHODS

### Cell culture and siRNA interference

BGC-823 and SGC-7901 cells were cultured with RPMI-1640 medium (Gibco, USA) supplemented with 10% fetal bovine serum (Gibco, USA) in 5% CO2 atmosphere at 37°C. Sp1 siRNA was purchased from Sigma Company and the catalog number is EHU018231. Chemical modified Stealth^™^ RBP2 siRNA was got from invitrogen and all the siRNAs were transfected with Lipofectamine 2000 (Invitrogen, Carlsbad, CA, USA) according to the protocol. The sequences for RBP2 and its control siRNA were 5'- CCA GCA CCA CCU CCU UCC UUC AUA A -3' and5'- CCU ACA UCC CGA UCG AUG AUG UUG A -3' respectively.

### Plasmid transfection and Luciferase reporter gene assay

Wild-type (WT) cagA/pcDNA3.1(+) plasmid (WT-cagA) was kindly provided by Zhu Yongliang (Zhejiang University, China) and this plasmid was described previously. CagA plasmid and its control plasmid which was purchased from Invitrogen were transfected with Roche Transfection Reagent (Roche, USA) according to the protocol. PGL- RBP2 promoter plasmid and PGL-TK were co-transfected into GC cells. The cells were lysed 48 hours later, mixed with the dual luciferase assay reagent (Promega, USA). Relative luciferase activity was calculated by normalizing the firefly luminescence as to the renilla luminescence.

### RNA extraction, RT-PCR and real-time PCR

Total RNA from cells were extracted with Trizol Reagent according to the protocol. The extracted RNA was then reverse-transcribed with RevertAid First Strand DNA Synthesis (RT) kit (Fermentas, life science, Canada). The cDNAs were used as the templates to amplify products using PCR method. The primers used were shown in table 1.

**Table 1:** primers for PCR

Genes	Primers
RBP2	5-GCTGCTGCAGCCAAAGTTG-3 (forward)
	5-AGCATCTGCTAACTGGTC-3 (reverse)
Cyclin D1	5-ATGGAACACCAGCTCCTGTG-3 (forward)
	5-ACCTCCAGCATCCAGGTGGC-3 (reverse)
Sp1	5-GGCGAGAGGCCATTTATGTGT-3 (forward)
	5-TGCATGACGTTGATGCCACT-3 (reverse)
AKT1	5-AGCGACGTGGCTATTGTGAA-3 (forward)
	5-ACAGTCTGGATGGCGGTTG-3 (reverse)
β-actin	5'-AGTTGCGTTACACCCTTTCTTG-3 (forward)
	5'-CACCTTCACCGTTCCAGTTTT-3 (reverse)

### Protein extraction and Western blot

Cells were lysed in protein lysis buffer and the proteins were subjected to quantity determination. Then the proteins were resolved on SDS-PAGE. The proteins in denaturing acrylamide gels were transferred to PVDF membrane and non-specific antigens were blocked by 5% nonfat milk. Primary antibodies for RBP2 (Abcam USA), β-actin (Sigma, USA), Sp1 (Cell Signaling, USA), CagA (Abcam USA), Cyclin D1 (Bioss, China), AKT (Bioss, China) and p-AKT (Bioss, China) were used to incubate overnight at 4°C and then immunoblot detection was performed with chemiluminescence (Millipore, USA) according to the protocol.

### Clinical specimen

36 samples of SG, AG with metaplasia and dysplasia respectively were obtained from Bengbu Medical University, Anhui province. The specimen were collected immediately after surgery and stored at formalin. The diagnosis of disease for all patients was confirmed by histological examination. General characteristics of patients were shown in table 2.

**Table 2:** Summary of characteristics of the patients

Case	Total samples	Male	Female	Age (range)	Age (median)
Superficial gastritis	36	22	14	21-72	51
Atrophic gastritis	36	26	10	43-80	57
dysplasia	36	26	10	21-78	60

### IHC

Sections from patients or mice were subjected to deparaffinating and dehydration. After antigen retrieve, H_2_O_2_ treatment and non-specific antigens blocking, the slides were incubated with monoclonal rabbit anti-human RBP2 (Sigma, USA; 1:150) or monoclonal rabbit anti-human Sp1 (Cell Signaling, USA) or rabbit anti-human Cyclin D1 (Bioss, China) overnight at 4°C. Secondary antibodies were used to incubate the slides and the avidin-biotin-peroxidase method was used to detect the antibody binding with DAB staining (Vector Laboratories, Burlingame, CA, USA).

### Construction of lentiviral vectors

BGC-823 cells with stable knockdown were generated using short hairpin RNAs (shRNA) directed against human RBP2 gene constructed in pLV3 vector got from GenePharma (Shanghai, China). Control cells were created by using a plasmid carrying nontargeting control sequence.

### EMSA

DIG Gel Shift Kit (Roche, USA) was used to label the following RBP2 promoter sequence: 5-AGAGGCGGGTAAGAGGCGG GTAAGAGGCGGGTA-3 and the experiment was performed according to the protocol provided. To be specific, 4 ul binding buffer, 1ul poly [d(I-C)] [1μg/μl], 1ul poly L-lysine[0.1 μg/μl] were added into nuclear extracts from GC cells and then were incubated with DIG-labeled probes in a 20ul reaction volume. The nuclear extracts were incubated with 400ng of antibody for super shift assay. 6% polyacrylamide gel was used to electrophorese the samples and then they were transferred onto a nylon membrane and ultraviolet-crosslinked. The DIG-labeled probe was detected with CSPD (Roche, USA) chemiluminescence reaction.

### ChIP

For ChIP assay, the kit (millipore, USA) was used to treat the prepared GC cells according to the protocol provided. The consequent precipitated DNA samples were detected with PCR method. The primes used in this experiment were the following: RBP2 promoters5'- GTGTAAAAGGAAAAGCAATTCATGG-3'(Forward);5'-AGGGTCCCTTTCCCTTTTGTG-3' (Reverse).

### Clone formation

After cells were subjected to the corresponding treatments, they were seeded in 6-well plates (300 cells/well), incubating for 15 days. Thereafter, 6-well plates were fixed with methanol and then were stained with Giemsa for 10 minutes and the number of colonies with more than 50 cells was counted for following analysis.

### Animal experiments

20 male nude mice were purchased from QING ZI LAN Animal Company (Nanjing, China) and divided into 2 groups. The mice were 7 weeks old and were subcutaneously injected with BGC-823 cells, 2×10^5^ cells/mouse. One group were injected into RBP2 shRNA stable-transduction cells and the other group were injected into the matched control cells. The tumors formed were measured every 3 days and all the mice were sacrificed 15 days later. Subcutaneous tumors were harvested, photographed and weighed, then the tumor index can be calculated.

### Statistical analysis

Data we got from biological replicates were presented as means (±SD or SEM). Student's t test was used to analyze the differences between different groups. P < 0.05 was considered to be significant.

## SUPPLEMENTARY FIGURE


