# Supplementary material for: Histone demethylase RBP2 induced by Helicobactor Pylori CagA participates in the malignant transformation of gastric epithelial cells
Source: Oncotarget. 2014 Jul 8;5(14):5798–807. doi: 10.18632/oncotarget.2185 (PMC4170602; doi:10.18632/oncotarget.2185)
Supplement: Supplementary file 1 [file oncotarget-05-5798-s001.doc]

Histone demethylase RBP2 induced by Helicobactor Pylori CagA participates in the malignant transformation of gastric epithelial cells

**Figure S1: (A)** RBP2 protein expression in GC cell lines using Western Blot. **(B)** RBP2 and Sp1 changes in RNA level with Sp1 siRNA and CagA treatment solely or jointly in GC cells using Semi-quantitative PCR. **(C)** The sequence (GGCGGG) in RBP2 promoter that can be bound by Sp1.
